# Androgen Concentrations in Umbilical Cord Blood and Their Association with Maternal, Fetal and Obstetric Factors

**DOI:** 10.1371/journal.pone.0042827

**Published:** 2012-08-20

**Authors:** Jeffrey A. Keelan, Eugen Mattes, HaiWei Tan, Andrew Dinan, John P. Newnham, Andrew J. O. Whitehouse, Peter Jacoby, Martha Hickey

**Affiliations:** 1 School of Women's and Infants' Health, King Edward Memorial Hospital, University of Western Australia, Perth, Western Australia, Australia; 2 Telethon Institute for Child Health Research, Centre for Child Health Research, University of Western Australia, Perth, Western Australia, Australia; 3 CPR Pharma Services Pty Ltd, BioSA Incubator, Thebarton, South Australia, Australia; 4 Department of Obstetrics and Gynaecology, University of Melbourne, The Women's Hospital, Melbourne, Victoria, Australia; Fudan University, China

## Abstract

The aim of this study was to measure umbilical blood androgen concentrations in a birth cohort using a highly specific liquid chromatography-tandem mass spectrometry (LC-MS/MS) assay and assesses the effects of sex, labor, and gestational age on fetal androgen levels at birth. We performed a prospective cohort study of androgen concentrations in mixed arterial and venous umbilical cord serum from 803 unselected singleton pregnancies from a general obstetric population in Western Australia. Total testosterone (TT), Δ4-androstenedione, and dehydroepiandrosterone were extracted from archived cord serum samples and measured using LC-MS/MS. SHBG was measured by ELISA; free testosterone (FT) and bioavailable testosterone (BioT) values were also calculated. Median values for all three androgens were generally lower than previously published values. Levels of TT, FT, BioT, and SHBG were significantly higher in male verses female neonates (P<0.0001), while dehydroepiandrosterone levels were higher in females (P<0.0001). Labor was associated with a significant (∼15–26%) decrease in median cord blood TT and FT levels (both sexes combined), but a modest (∼16–31%) increase in SHBG, Δ4-androstenedione, and dehydroepiandrosterone concentrations. TT and FT were significantly negatively correlated with gestational age at delivery, while SHBG, Δ4-androstenedione, and dehydroepiandrosterone were positively correlated. Antenatal glucocorticoid administration also had a significant effect in the multiple regression models. This is the first study to report umbilical cord androgen levels in a large unselected population of neonates using LC-MS/MS. Our findings suggest that previous studies have over-estimated cord androgen levels, and that fetal, maternal, and obstetric factors influence cord androgen levels differentially. Caution should be exercised when interpreting previously-published data that have not taken all of these factors into account.

## Introduction

Fetal androgen concentrations have been the subject of considerable interest over several decades due to their association with maternal metabolic disorders [1]–[3], later life cancer risk [4]–[6] and reproductive [7]–[12] and behavioral/neurodevelopmental problems [13]–[18]. Androgen concentrations in umbilical cord blood have been widely used as a marker of prenatal androgen exposure, with most published studies using radioimmunoassay (RIA) to measure steroid concentrations [1], [3], [5], [8], [11], [19]–[26]. More robust studies have used organic solvent extraction to remove interfering factors in order to improve specificity, accuracy and sensitivity. A few have employed additional column purification techniques [6], [27]–[29] to address concerns regarding the accuracy of RIAs for the measurement of low concentrations of testosterone [30], [31]. However, while androgen assays are sometimes tested and validated for use in female or pediatric samples, their suitability for umbilical cord blood analysis is usually unverified. Cord blood contains an abundance of unusual and potentially cross-reacting steroids and their conjugates, in addition to other interfering substances [32], [33]; hence, it is critical that steroid assays are properly validated prior to use in the analysis of cord blood.

Increasing awareness of the limitations of RIA for the measurement of low concentrations of sex steroids [30], [34], [35] has led to the adoption of mass spectrometry as the preferred methodology for the measurement of circulating testosterone levels in women and children, with reported concentrations consistently lower than those derived by RIA [35]–[39]. To the best of our knowledge, only three studies have been published in which mass spectrometry has been applied to cord blood androgens measurements [7], [33], [36]. In one of these studies, assay sensitivity was a significant limitation (7), while only two of these studies described cord blood concentrations of the weak androgens Δ^4^-androstenedione (A4) and dehydroepiandrosterone (DHEA) [7], [33]. However, irrespective of assay methodology, previously published studies of cord androgens have suffered from a lack of power [6], [7], [17], [19], [33], or insufficient control over potential fetal and obstetric confounders such as fetal sex [20], [33], presence and duration of labor [7], [22], [36], [38], gestational age at delivery and maternal factors such as ethnicity, age, parity and smoking status [5], [7], [20], [24], [26], [40]. Fetal adrenal steroid production changes with gestational age and labor, while levels of steroid metabolizing enzymes in the placenta are regulated by factors known to be associated with labor and delivery such as glucocorticoids, pro-inflammatory cytokines and exposure to reactive oxygen species [41]. Hence, it is highly likely that factors such as prematurity, labor onset, placental weight, intrauterine infection and preeclampsia could influence umbilical cord androgen levels, although the nature and extent of their influence has not yet been determined.

To address the weaknesses of previous studies and provide robust data on the relationships between obstetric variables, fetal sex and cord androgens, we have (as far as we are aware) undertaken the largest study to date of umbilical cord serum androgen concentrations determined by liquid chromatography-tandem mass spectrometry (LC-MS/MS). The size of the cohort, combined with the accuracy and validity of the LC-MS/MS method, provides for the first time a robust and accurate assessment of the relationships between fetal sex, obstetric variables, gestational age at delivery and fetal androgen levels at birth.

## Methods

### Cord blood samples

The Western Australian Pregnancy Cohort (known as the Raine Study: www.rainestudy.org.au) consists of 2868 unselected pregnancies recruited and sampled from the public antenatal clinic of King Edward Memorial Hospital in Perth, Western Australia between 1989–91 to study the effects of repeated ultrasound on fetal and post natal growth & development and pregnancy outcome. Study participants completed a questionnaire at 18 and 34 weeks gestation, which provided information on a wide range of variables including ethnicity, social and economic circumstances, lifestyle, medical history and environmental exposures. Pregnancy and neonatal outcome data were taken from hospital notes. The study was approved by the King Edward Memorial Hospital/University of Western Australia ethics committee and participants were re-consented in writing at 18 years of age. Umbilical cord blood was collected from 870 deliveries from the intensive ultrasound arm of the cohort, which consisted of 1415 singleton pregnancies in total. Immediately after delivery mixed umbilical arterial-venous (UA∶UV) cord blood was collected, allowed to clot and the resulting serum was frozen at −80°C and stored without thawing until the present study was performed. Detailed sequence analysis of DNA obtained from ten maternal-child pairs confirmed that the cord blood samples were not contaminated by maternal blood. Eight hundred and three cord blood samples (92.3%) representing 407 male and 396 female infants had sufficient serum (after removal, aliquoting and archiving of 1 ml for future studies) for steroid analysis. The demographic and obstetric characteristics of the sample set are shown in **Table 1**. Cord serum samples were thawed, aliquotted and shipped from Perth to South Australia for LC-MS/MS analysis (CPR Pharma Services Pty Ltd, Thebarton, SA); in total samples were thawed and frozen less than three times following collection.

**Table 1:** Demographic and obstetric characteristics of the study cohort by fetal sex.

	Males	Females	Total^g^	*P*
	n = 407	n = 397	n = 803	
Maternal age, years^a^ (total)	28.2±6.1	27.9±6.0	28.0±6 (803)	NS^h^
Multiparous, % (total)	51.0	55.3	53.1 (803)	NS
Low income family^b^, % (total)	60.9	53.4	57.2 (731)	0.044
Caucasian ethnicity, % (total)	89.7	84.8	87.3 (802)	0.044
Smoker^c^, % (total)	24.0	25.5	24.8 (727)	NS
Gestational age at delivery, weeks^a^ (total)	39.1±2.2	39.2±2.1	39.2±2.1 (791)	NS
Birthweight, kg^a^ (total)	3.35±0.62	3.28±0.56	3.32±0.59 (800)	0.018
Placental weight, g (total)	582.3±125.7	591.3±123.2	586.9±124.5 (801)	NS
Instrumented delivery, % (total)	41.0	37.5	39.4 (803)	NS
Cesarean section^d^, % (total)	18.9	17.9	18.8 (803)	NS
Delivery post-labor^e^, % (total)	88.7	90.2	89.5 (803)	NS
Labor duration, h^a^^,^ f (total)	6.5±4	7.2±4.6	6.9±4.3 (657)	NS
Prelabor rupture of membranes, %	20.5	17.9	19.2 (801)	NS
Induced labor, % (total)	25.2	27.8	26.5 (801)	NS
Antepartum hemorrhage, % (total)	8.1	8.8	8.5 (801)	NS
Tocolysis administered, % (total)	1.2	0.5	0.9 (801)	NS
Antenatal steroids administered	3.2	2.5	2.9 (801)	NS
Preterm birth, % (total)	7.4	7.3	7.4 (801)	NS
IUGR, % (total)	3.5	3.0	3.2 (801)	NS

a Mean+/−SD;

b income <$24,000 per annum;

c smoking defined as one or more cigarettes per day;

d includes both elective and in-labor sections;

e all deliveries excluding elective Cesarean-section deliveries;

f stage 1 and 2 labor combined;

g the number of pregnancies included for each category or variable are indicated in parentheses;

h NS,

not significant (*P*>0.05 by Mann-Whitney U-test for group median comparisons or Fisher's Exact test for categorical variables).

### Steroid analysis

Total testosterone (TT), Δ^4^-androstenedione (A4), dehydroepiandrosterone (DHEA), and internal standards (d3-testosterone [IS-T] and 19-d3-androstenedione [IS-A4]) were extracted from 20 µL aliquots of cord serum with hexane∶dicholoromethane (3∶2). Steroid extracts were separated by HPLC on a Kinetex C18 column with a run time of 10 minutes, and the eluates monitored by an API5500 Q-TRAP MS/MS detector in positive MRM mode. All three steroids were well resolved chromatographically; the single charged Q1/Q3 transitions for TT, A4, and DHEA were 289.3/96.9, 287.1/97.0, and 289.2/253.2 atomic mass units (amu), respectively, and 292.2/97.0, and 290.2/100.0 amu for IS-T and IS-A4, respectively. The data were acquired and processed by the data acquisition system Analyst® linked directly to the API5500 Q-TRAP MS/MS detector. Calibration curves (0.05–10.0 ng/mL for TT, 0.10–20.0 ng/mL for A4 and 1.00–200 ng/mL for DHEA) were prepared in stripped human serum and were run with each assay batch; calibration slope regression coefficients were ≥0.996 (n = 12). Recovery was calculated using deuterated internal standards added to the samples prior to extraction. The limits of quantitation for TT, A4 and DHEA were 0.025, 0.05 and 0.05 ng/ml, respectively (0.08, 0.17 and 0.17 nmol/L). Extensive optimization and validation was carried out using fresh cord serum to ensure that the assays were specific and accurate. Quality controls (stripped serum) at low, medium and high values demonstrated inter-assay imprecision (coefficient of variation, CV) of 5–8%, 5–14% and 8–11% for TT, A4 and DHEA, respectively (n = 24). Pooled male and female cord serum QC samples were also run with each batch and performed with comparable precision: CVs for TT, A4 and DHEA were 6–11%, 7–8% and 8–18%, respectively (n = 24). Recovery of target analytes from stripped serum across three concentrations ranged from 93–98% (TT), 104–111% (A4) and 93–96% (DHEA). Assay performance was determined to be unaffected by up to three freeze-thaw cycles or 24 h at room temperature. Steroid analysis was performed blind to sample identity or characteristics.

Sex hormone binding globulin (SHBG) was measured by ELISA using a commercial kit (IBL International, Hamburg, Germany) according to the manufacturer's instructions. All samples were measured in duplicate by a single operator using assay kits from the same batch. Samples with an initial replicate CV of >10% were reanalyzed. The inter-assay imprecision (CV) was <4.5% (n = 25); intra-assay CV was 5.2% (n = 861).

### Calculation of free testosterone (FT) and bioavailable testosterone (bioT)

The mass action equation method of Vermeulen et al [42], which is frequently used to calculate FT, is not recommended for samples with atypical albumin, SHBG and steroid profiles, and has recently been shown to overestimate the true FT by 40–60% [43]–[45]. Therefore, we employed an empirical method for calculating FT, validated for use in samples with low TT and SHBG concentrations, as described by Sartorius et al [45]:
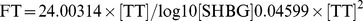
A comparison of FT values derived using the Vermeulen method (via the online calculator: http://www.issam.ch/freetesto.htm) *vs*. the empirical method carried out in n = 25 samples selected across a range of TT and SHBG values revealed that the empirical method generated values that were on average 32% lower than those derived by the Vermeulen method, consistent with expectations [42]. BioT values were calculated using the standard formula: BioT = [FT]+[albumin-bound T] with K_alb_ equal to 36,000 L/mol [42]. Previous studies of androgen levels in umbilical cord blood have calculated BioT values based on the assumption that all samples had equal albumin concentrations, despite the fact that albumin levels decrease with gestational age [46], [47]. To take the effect of gestational age into account albumin levels were derived for each week of gestation, ranging from 19.5 g/L at 29 weeks to 30 g/L at term, based on published reference data [46], [47], and these values were used in the BioT calculation.

### Statistical analysis

Descriptive statistics were prepared on Microsoft Excel. All sets of continuous data failed the normality test of Kolmogrov and Smirnov so group medians were compared by Mann-Whitney U-test using Instat3 (GraphPad Software Inc, La Jolla, CA). Categorical or discrete variables were compared by Fisher's Exact Test. A *P* value<0.05 was considered significant. Linear correlations were performed on Instat3 to explore the associations between androgen levels, gestational age at delivery, birth weight and placental weight after removal of extreme outliers (TT, n = 2; A4, n = 1; DHEA, n = 2; FT, n = 4; BioT, n = 4); there were no significant departures from linearity. Multiple regression analyses were then performed (using Instat3) to determine the independent effects of a range of maternal and obstetric variables (maternal age, parity, ethnicity, socioeconomic status, labor onset/duration, prelabor rupture of membranes [PROM], antepartum hemorrhage [APH], labor induction, administration of tocolytics, smoking status, antenatal glucocorticoid exposure, preterm birth, preeclampsia/hypertension [PE], prelabor rupture of membranes [PROM], intrauterine growth retardation [IUGR], birthweight, placental weight and gestational age at delivery) on cord blood androgen concentrations and their associations.

## Results

The demographic details of the entire cohort, segregated according to fetal sex, are shown in **Table 1**. Male neonates were, on average, slightly heavier (67 g) than females, as expected. In addition, mothers with male fetuses were significantly more likely to be from a low income background and of Caucasian ethnicity. There were no differences in maternal age, parity, smoking status, gestational age at delivery, labor duration, placental weights, rates of instrumental delivery, Cesarean section, PROM, APH, PE, tocolytic administration, preterm birth or IUGR (Table 1).

**Figure 1:**
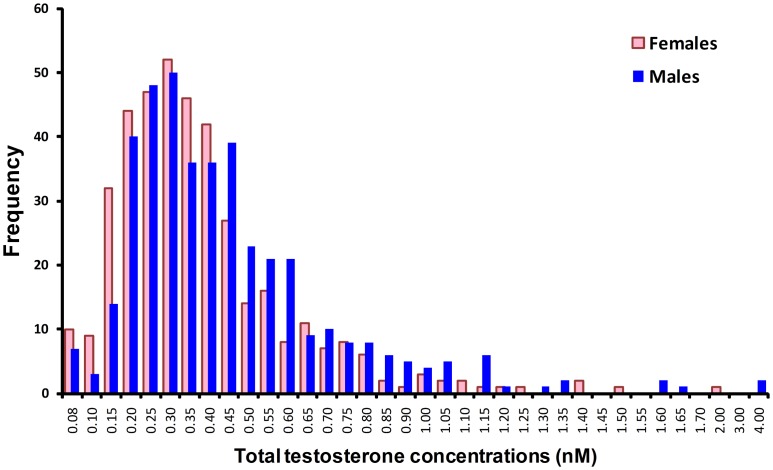
Frequency distribution of total testosterone (TT) values in male and female cord blood samples (n = 803).

Of the 803 cord blood samples assayed, concentrations of TT and DHEA were below the limits of quantitation (0.08 and 0.17 nM, respectively) in 17 and 8 samples, respectively. These samples were assigned values at the LOQ for statistical analysis. Two samples were not assayed for SHBG. The mean, SD, median and range for all four analytes including the calculated FT and BioT values in male and female neonates are shown in **Table 2**. Median concentrations of TT were significantly (62%) higher in males compared to females (*P*<0.0001), as were FT and BioT levels (55–71%, *P*<0.0001); median SHBG concentrations were also modestly elevated in males (10.6%, *P*<0.0001). In contrast, DHEA concentrations were significantly higher (19.5%) in females verses males (*P*<0.0001). There were no significant differences in A4 concentrations. The distribution of all three androgens was skewed to the right of the median due to the presence of a number of values with elevated values (**Figure 1**). This skewness was unrelated to birthweight, placental weight, or any other variable tested. Analysis of the distribution of values in males and females indicated that in 10.5% of males, cord TT concentrations were higher than 0.75 nmol/L, compared to only 5.8% of females. In contrast, only 15.7% of male neonates had TT values <0.2 nmol/L compared to 24.0% of females.

**Table 2:** Total testosterone (TT), Δ^4^-androstenedione (A4), dehydroepiandrostenedione (DHEA) and SHBG concentrations, plus calculated free testosterone (FT) and bioavailable testosterone (BioT) levels in umbilical cord serum samples from male and female neonates.

		TT	A4	DHEA	SHBG	FT	BioT
		nmol/L	nmol/L	nmol/L	nmol/L	pmol/L	nmol/L
Males	Mean+/−SD	0.50+/−0.34	2.26+/−1.18	7.46+/−6.89	24.22+/−15.06	9.60+/−7.90	0.14+/−0.1
	Median	0.42	2.04	6.38	21.40	7.50	0.12
	(range)	(0.08–3.78)	(0.49–16.06)	(0.2–112.3)	(0.60–204.9)	(1.58–84.13)	(0.02–1.06)
Females	Mean+/−SD	0.30+/−0.20	2.28+/−0.91	9.44+/−10.31	20.91+/−11.50	5.89+/−4.77	0.09+/−0.07
	Median	0.26	2.18	7.63	19.35	4.85	0.07
	(range)	(0.08–2.19)	(0.45–6.77)	(0.2–184.8)	(2.6–118.3)	(1.15–65.34)	(0.0–1.02)

Concentrations of all analytes were significantly different between the sexes (*P*<0.0001, Mann-Whitney test) with the exception of A4.

**Figure 2:**
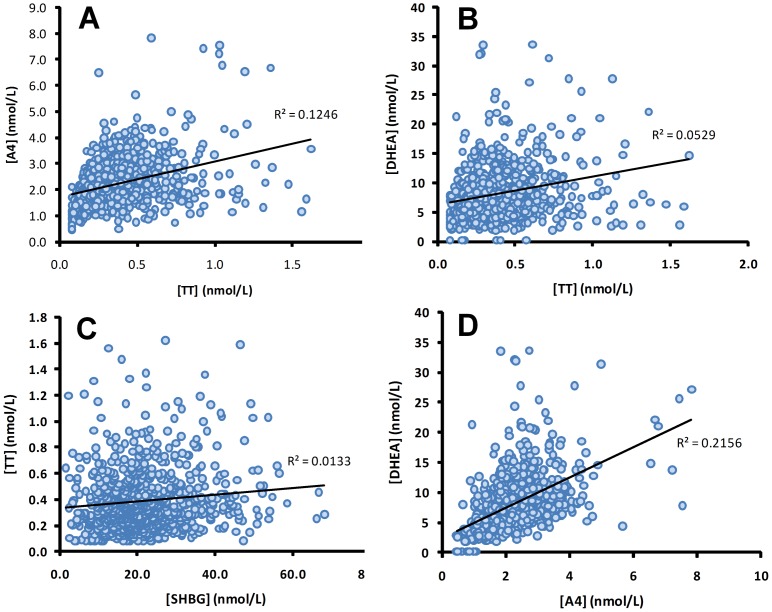
Linear regression analysis of cord androgen and SHBG concentrations in 803 neonates (males and females combined). The linear regression coefficients (R^2^) are indicated on each graph. All correlations were statistically significant (A, B and D: *P*<0.0001; C: *P*<0.0005) and remained significant after controlling for multiple maternal and obstetric factors in multiple regression analysis.

Linear regression and multiple regression analyses were performed to assess the inter-relationship between androgen and SHBG levels, controlling for the maternal and obstetric variables listed in Table 1; the data were analysed with males and females combined as segregation by sex did not influence the correlations. Overall, TT concentrations were weakly, but significantly, correlated with those of A4 (r^2^ = 0.125, *P*<0.0001), DHEA (r^2^ = 0.053, *P*<0.0001) and SHBG (r^2^ = 0.016, *P*<0.0005) (**Figure 2**). Significant correlations were also seen between SHBG and A4 (r^2^ = 0.022, *P*<0.0001; data not shown) and more strongly between A4 and DHEA (r^2^ = 0.215, *P*<0.0001). All correlations remained highly significant (*P*<0.0001) after controlling for ethnicity, socioeconomic status, birth weight, placental weight, gestational age at delivery, parity, tobacco smoking status (0, 1–10 or >11 cigarettes smoked per day), labor onset, labor induction, labor duration, oxytocin administration, tocolytic administration, antenatal steroid administration, APH, PTB, and PROM in multiple regression analysis. Maternal age showed a weak but significant negative correlation with cord blood A4 concentrations (r^2^ = 0.016, *P* = 0.0004), but not with any of the other endocrine measurements; this association remained significant in the multiple regression analysis.

**Figure 3:**
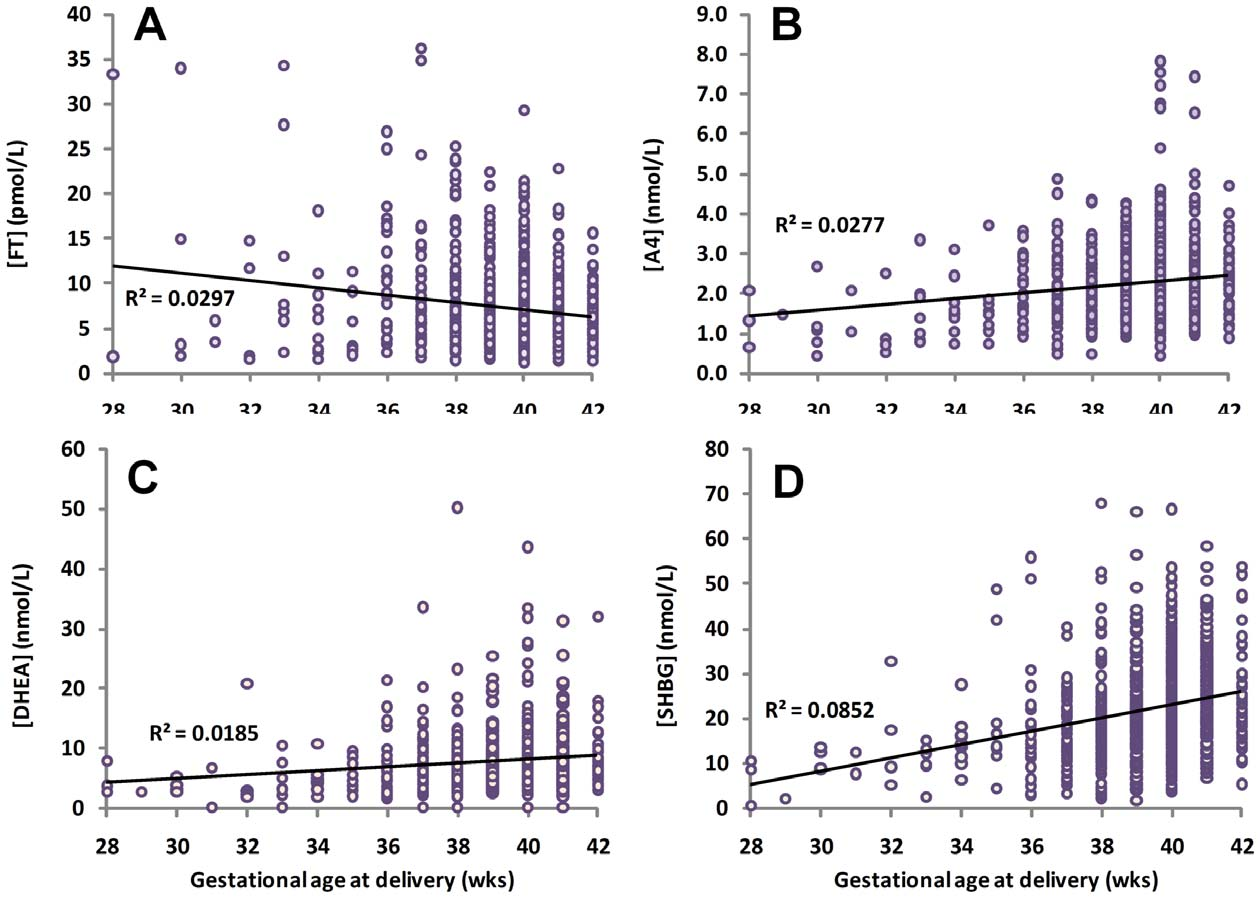
Linear regression analysis of the relationships between gestational age at delivery and cord blood androgen and SHBG concentrations in 803 neonates (males and females combined). The linear regression coefficients (R^2^) are indicated on each graph. All correlations remained statistically significant (*P*<0.0001) after controlling for obstetric factors.

**Figure 4:**
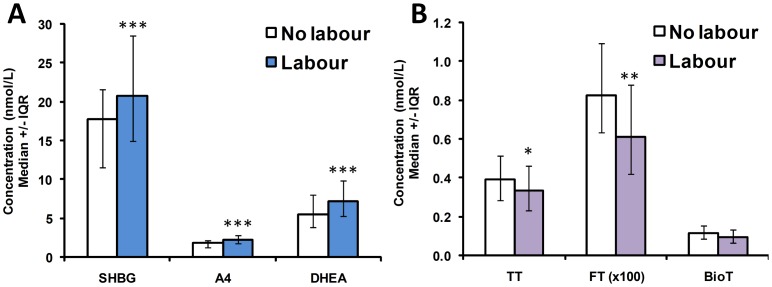
Effects of labor on androgen and SHBG concentrations. A) SHBG, A4 and DHEA concentrations are significantly increased with labor (n = 83 no labor, 718 with labor); B) TT and FT, but not BioT, levels are significantly reduced with labor. Data shown are median ± interquartile range (IQR). Note that the FT values have been multiplied by 100 to allow presentation on the same graph. *, *P*<0.05; **, *P*<0.01; ***, *P*<0.0005 by Kruskal Wallis test.

Multiple regression analysis also revealed a significant effect of gestational age at delivery on the association between SHBG levels and A4 and DHEA (*P*<0.0001), with weaker effects between SHBG and TT, fT and BioT (*P* = 0.0002). Linear correlations demonstrating a positive correlation between gestational age and SHBG, A4 and DHEA concentrations (*P*≤0.0001)), and a weak negative correlation with TT and FT concentrations (*P* = 0.0425 and *P*<0.0001, respectively), are shown in **Figure 3**. Duration of labor (stage 1 and 2 combined) was weakly correlated with SHBG concentration (r^2^ = 0.014, *P* = 0.0019) but this did not remain significant after adjusting for other obstetric variables. On the other hand, delivery following the onset of labor compared to delivery by elective Cesarean section prior to labor had a significant effect on hormone levels in the multiple regression models. Group comparisons revealed that median SHBG, A4 and DHEA concentrations were increased with labor by 16.3%, 24.4% and 30.8%, respectively (*P*<0.0005) (**Figure 4A**). In contrast, TT and FT were reduced by ∼15–26% with labor (**Figure 4B**); BioT levels were similarly lower but this did not reach statistical significance. The presence of PE had a weak effect in some multiple regression models, but group comparisons found no evidence of any differences in androgen or SHBG levels with PE (**Figure S1A**). Administration of antenatal glucocorticoids was also shown to have a significant effect on the associations between SHBG, TT, FT, BioT, A4 and DHEA levels in multiple regression analysis after adjusting for gestational age at delivery; however, a comparison of androgen levels in samples delivered with and without glucocorticoid administration did not find any significant differences between the groups, although mean levels of TT, FT, BioT and DHEA were somewhat higher in the steroid group (**Figure S1B**).

## Discussion

There has been a recent surge of interest in the effects of prenatal androgen exposure on postnatal cognition, behaviour and health. This study of umbilical cord androgen concentrations measured by LC-MS/MS in an unselected population of male and female neonates is the largest to date and provides for the first time robust data on the relationship between fetal and obstetrics factors and circulating androgen and SHBG levels at birth. Umbilical cord androgen concentrations have been measured previously in a number of smaller studies using immunoassay methods [1], [3], [5], [8], [11], [19]–[26]. However, it is now widely appreciated that accurate measurement of low concentrations of androgens (testosterone in particular) using immunological techniques is fraught with difficulties relating to assay sensitivity and specificity limitations [31], [34]. This has led to the increasing adoption of mass spectrometry for generation of reliable sex steroid data in fluids from women and children [30], [37]. To the best of our knowledge, only three previous studies have applied this technology to the measurement of steroid levels in cord blood, none of which investigated the influence of key fetal or obstetric factors. Our study, which is by far the largest study of cord blood androgens to date, employed solvent extraction to remove interfering substances and a stringent and lengthy chromatographic separation to ensure that the steroids of interest were well resolved prior to MS/MS detection. Our own in-house comparisons (data not shown) revealed dramatic differences in TT values between an extraction RIA and our LC-MS/MS results; such differences, however, will be entirely assay dependent. Cord serum represents a particularly challenging medium to analyze as it has an unusual steroid profile due to the unique combination of placental and fetal steroid biosynthesis and metabolism (conjugation and deconjugation) and so assays require particularly careful validation to ensure that the results are accurate [32], [33]. Due to the presence of a variety of steroid isomers in cord blood with almost identical fragmentation patterns, efficient chromatographic discrimination of analytes is particularly important.

While the size of the cohort and use of LC-MS/MS is a strength of our study, a potential weakness is the age of the samples (∼19 years). The major concern is that degradation may have occurred in storage or with thawing, resulting in reduced concentrations of intact steroids. However, we believe such concerns are unfounded for several reasons. Firstly, studies of steroid stability (including testosterone) during long term storage confirm that serum samples are able to be stored for at least four decades at −80°C without loss or appreciable deterioration of steroid hormones [48], [49]. Our sample set had been continually maintained at −80°C since collection and samples were thawed only once for aliquotting prior to shipping for assay. Secondly, freshly collected cord blood samples run as QCs (n = 5–6) had values within one standard deviation of the means of the frozen samples, adding further weight to the view that no significant degradation had taken place; our stability studies showed no effects of several freeze-thaw cycles. Finally, our recovery estimates based on spiking of fresh cord serum were 93–111% indicating that the assays did not suffer from masking or interference effects. Therefore, we conclude that our results are accurate and precise, and differences between our values and those of previous studies likely reflect either a lack of selectivity of the assays used in other studies, confounding sample variables, or small sample size.

An additional potential limitation of our study is the use of mixed UA∶UV blood samples. While we have confirmed that our samples are not contaminated by maternal blood, the relative proportion of umbilical arterial and venous blood is unknown and, in theory, may be altered by factors such as mode of delivery, gestational age, placental architecture and fetal hemodynamics. Studies which have compared UA∶UV differences in cord blood steroids suggest that UV concentrations of TT, A4 and DHEA are approximately 20%, 40% and 50–75% lower than UA levels [20], [33], representing the differential effects of placental metabolism. Hence, variations in relative proportions of UA and UV blood could have significant confounding effects which we are unable to control for in this study. It should be noted that most large cohort studies would also suffer from this limitation.

While we anticipated that our results would be marginally lower than many previous studies as a result of improved assay accuracy and precision, we were surprised that the median TT values for our cohort were markedly lower than published values, even some of those derived by mass chromatographic methods. For instance, Krogh and colleagues recently reported TT values in the range of 0.8–0.9 nmol/L in a LC-MS/MS study of 10-year-old cord blood samples from approximately 200 male infants from the Danish National Birth Cohort [36]; TT was also measured in 9 control female infants and averaged 0.5 nmol/L. These values are similar to those reported by Troisi et al using a robust and well validated RIA [6], [27], [29], and are considerably (<80%) lower than those described in many other studies using less stringent assays [5], [8], [11], [17], [22], [26], but are nevertheless markedly higher than the values we report here. Anderson *et al.* reported mean TT values of 0.5 nmol/L in seven male infants measured by LC-MS/MS, in keeping with our results, although half were below the limit of detection of 0.24 nmol/L [7]. Hill *et al.*, in a GC-MS study of the umbilical blood steroid metabolome [33], described the concentrations of 40 unconjugated steroids in UA and UV plasma from 12 infants of undefined sex delivered at term following normal labor, and 38 neonates delivered preterm (28–36 weeks gestation) with various complications. They reported TT values in UA and UV samples of 1.25 and 1.04 nmol/L, respectively, approximately double the values we report here in mixed UA∶UV samples. Unfortunately, in this study TT values were not segregated on the basis of fetal sex, gestational age or labor status. These investigators also reported mean UA∶UV concentrations of A4 and DHEA of ∼2.8 nmol/L and ∼5.5 nmol/L, respectively. These values compare reasonably well with our mixed UA∶UV levels (∼2.2 nmol/L and 8.0 nmol/L) and those of Laatikainen *et al.* [20], but are markedly lower than those reported by Troisi [6], [28], [29], [50] and others [7], [21], [23], [26]. The collective implication of these findings is that the majority of previously reported cord blood androgen measurements are confounded, to a greater or lesser degree, by significant cross-reactivity with undefined interfering material. Androgen data from previous studies should, therefore, be interpreted with considerable caution, and some of the resulting conclusions regarding the relationship between prenatal androgen exposure and postnatal development may need to be reexamined.

In agreement with several previous studies [26], [29], [36], [51], we reported significantly higher levels of cord blood TT in male verses female babies, although the difference was relatively modest (∼60%) which may explain the lack of difference observed by some investigators. However, we observed an interesting sex-dependent difference in the relative distribution of high and low outliers, with a preponderance of the samples with very high levels being males and those with low levels being females. Assuming these values are reflective of levels in utero earlier in gestation, this indicates that exposure to relatively high testosterone concentrations during fetal development is likely to be more common in male *vs.* females fetuses, whereas the converse is true with respect to relative androgen underexposure. This has important implications for studies investigating the relationships between the development of androgen-sensitive neurocognitive abnormalities or metabolic disorders and in-utero androgen exposure. It also emphasizes the need for developmental studies to be sufficiently powered to accommodate such ‘extremes’ in their analyses. We also described novel findings of higher DHEA concentrations in cord blood from female infants compared to males. Small differences have been observed in previous studies [6], [7], [29] but due to lack of power these were not deemed significant. In contrast, we found that cord A4 levels were similar between the sexes, unlike the study of van der Beek et al who reported higher values in male *vs.* female infants [26]. This discrepancy is most likely to due to differences in assay specificity. The explanation for our finding of higher DHEA levels in female neonates is unclear, but could reflect either greater fetal adrenal DHEAS production, increased placental DHEAS metabolism (desulfation), or decreased placental conversion of DHEA to either A4 and testosterone or estrogens (fetal DHEAS is the major substrate for placental estrogen production from 20 weeks pregnancy). Interestingly, we found no evidence of an effect of placental size on cord blood androgen levels, which might have been expected based on the known importance of placental mass on some endocrine parameters in pregnancy.

All three androgens measured in this study are substrates for estrogen biosynthesis via the actions of placental aromatase/CYP19 and are also inter-convertible via other placental enzymes [41]. This may explain the observation that levels of all three steroids were significantly correlated, despite the fact that the tissue origin of the three steroids varies. We also observed significant effects of labor on cord blood androgen levels. Labor is associated with increased fetal and maternal stress leading to elevated adrenal steroid (DHEAS) production. In addition, placental aromatase activity has been reported to decrease with labor [52], in which case placental conversion of androgens to estrogens should be reduced, again tending to result in increased fetal androgen levels. Several researchers have previously described increases in cord blood levels of A4 [21], [23] and DHEA [21], [53] with labor, speculating that this reflects increased production of C19 steroids by the fetal adrenal in association with a labor-induced HPA stress response. Our findings agree with these publications. However, in contrast to A4 and DHEA, we also showed that labor was associated with a decrease in cord TT concentrations together with an increase in SHBG levels, the combination of which results in a highly significant reduction in FT levels with labor. The biological significance and biochemical explanation for these novel observations are unknown. Nevertheless, despite the positive correlations observed between all three androgens in this study, it would appear that testosterone biosynthesis and metabolism can be differentially regulated during labor. Unfortunately, estrogen data on this cohort are not yet available so we cannot determine at this time whether the changes in androgen levels are mirrored by changes in their aromatization products. Estrogen analysis of the same sample set is currently underway. Somewhat surprisingly, we observed no evidence that antenatal glucocorticoid administration was associated with reduced cord blood androgen levels, which might be predicted as a result of fetal adrenal suppression. Indeed, if anything androgen levels tended to be higher in the pregnancies that received antenatal steroids, although our study was not powered to explore this issue definitively.

SHBG was measured primarily to allow determination of FT and BioT values. Unexpectedly, we uncovered some interesting associations between SHBG levels and fetal and obstetric factors. A highly significant positive relationship was observed between cord SHBG concentrations and gestational age at delivery which remained after controlling for multiple variables; this was described previously in a small study [54] but is now confirmed in our much larger cohort. Similarly, in a previous study of cord blood from 98 healthy newborns, males were reported to have higher SHBG levels than females [55], an observation which was corroborated in the present study. In order to calculate FT we used a relatively recent empirical method from the Handelsman laboratory [44], [45] since this method has been validated for use in samples with low TT concentrations and addresses the positive bias of the more orthodox mass action equation [42]. We also corrected for the gestational age-dependent changes in fetal albumin concentrations [46], [47] in the calculation of BioT to eliminate this source of additional inaccuracy. Due to limitations in sample volume were not able to measure albumin concentrations directly. Using these approaches we have generated values for FT and BioT in our sample cohort that are as accurate as possible in the absence of measurement by equilibrium dialysis. As expected, FT and BioT were strongly correlated with TT values; while they may be more meaningful measures of androgen exposure than TT, the validity of this view is open to debate.

Some studies suggest that smoking in pregnancy may increase circulating postnatal testosterone and cortical concentrations and reduce estriol concentrations [56], [57]. However, in our cohort we found no evidence of a significant relationship between tobacco exposure and cord serum androgen levels. We also found no evidence of a significant association between maternal age and TT levels, although A4 concentrations were negatively correlated with maternal age. A negative association between maternal circulating A4 levels and maternal age has been previously reported [58]; our data suggest that this unexplained phenomenon may encompass the fetal compartment also, although the biological implications, if any, remain to be determined. We also failed to find strong evidence of an association between preeclampsia and cord androgen levels, in agreement with the findings of Troisi and colleagues [50]. Similarly, IUGR did not significantly interact with any of the endocrine variables.

In conclusion, we report that levels of androgens in umbilical cord blood samples from unselected male and female neonates are lower than previously described, and that umbilical cord androgen levels are influenced by fetal sex, onset of labor and gestational age at delivery. We suggest that most previous studies examining the relationships between cord blood androgens and later-life/developmental events are compromised by inaccurate steroid measurements or inadequate control for fetal or obstetric factors. As such, some of their conclusions may be questionable and considerable caution should be exercised when interpreting previously-published data that have not taken these factors into account.

## Supporting Information

Figure S1 **Androgen and SHBG concentrations (nM, mean+/−SD) in cord blood samples from pregnancies with or without (A) preeclampsia/severe hypertension, or (B) maternal antenatal glucocorticoid administration.** Note: fT values were multiplied by 100 to allow representation on the same graph. No significant differences between groups were detected by Kruskal-Wallis test (P>0.05)
(TIF)
