# Supplementary figures and images for: Androgen Concentrations in Umbilical Cord Blood and Their Association with Maternal, Fetal and Obstetric Factors
Source: PLoS One. 2012 Aug 20;7(8):e42827. doi: 10.1371/journal.pone.0042827 (PMC3423422; doi:10.1371/journal.pone.0042827)

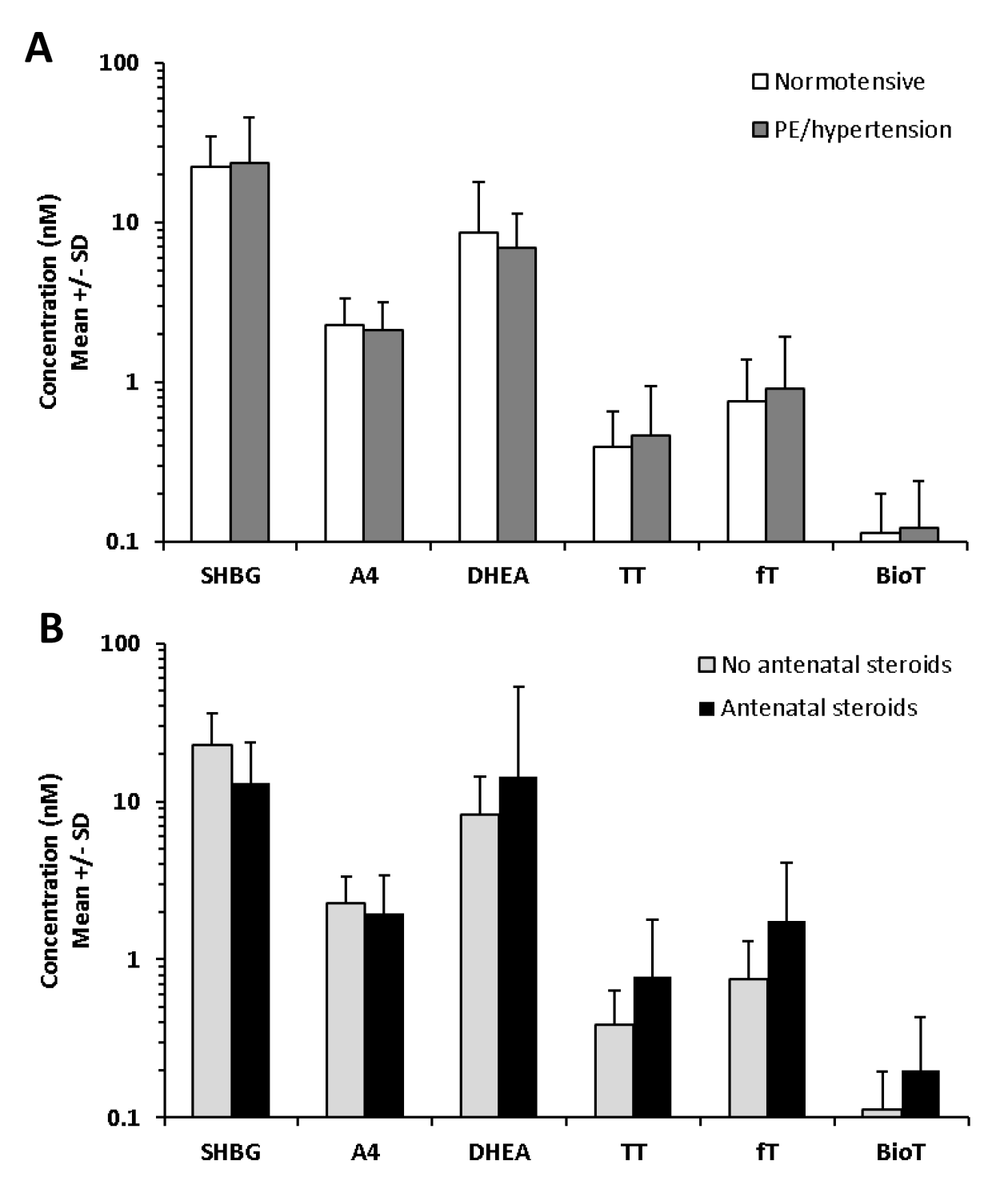

Supplement: Figure S1 — Androgen and SHBG concentrations (nM, mean+/−SD) in cord blood samples from pregnancies with or without (A) preeclampsia/severe hypertension, or (B) maternal antenatal glucocorticoid administration. Note: fT values were multiplied by 100 to allow representation on the same graph. No significant differences between groups were detected by Kruskal-Wallis test (P>0.05) (TIF) [file pone.0042827.s001.tif]
